# Diagnostic and Therapeutic Challenges in a Patient with Ureteral Metastases from a Triple Negative Breast Cancer

**DOI:** 10.3390/curroncol29070380

**Published:** 2022-07-07

**Authors:** Georgia Saranti, Vasiliki Zolota, Christina Kalogeropoulou, Nikolaos Papathanasiou, Theodora Katsila, Konstantina Kitsou, Ilias Haliassos, Dimitrios Kardamakis

**Affiliations:** 1 Department of Radiation Oncology, University Hospital of Patras, University Campus, 26504 Patras, Greece; zeta7068@gmail.com (G.S.); ehaliassos@gmail.com (I.H.); 2 Department of Histopathology, University Hospital of Patras, University Campus, 26504 Patras, Greece; zol@med.upatras.gr; 3 Department of Radiology, University Hospital of Patras, University Campus, 26504 Patras, Greece; ckrat@upatras.gr; 4 Department of Nuclear Medicine, University Hospital of Patras, University Campus, 26504 Patras, Greece; nikopapath@upatras.gr; 5 Institute of Chemical Biology, National Hellenic Research Foundation, 11635 Athens, Greece; thkatsila@eie.gr; 6 Department of Hygiene and Epidemiology, University of Patras Medical School, 26504 Patras, Greece; up1064765@upatras.gr

**Keywords:** triple negative breast cancer, ureteral metastases, imaging, radiotherapy

## Abstract

Metastatic ureteral tumors arising from a primary breast carcinoma are extremely rare. They present with hematuria and radiological findings compatible with obstructive ureteral phenomena. We present a case of an 87-year-old woman with a history of lymphoma and triple negative breast cancer (TNBC), during an emergency admission for peptic ulcer, developed macroscopic hematuria. Radiologic and endoscopic investigations revealed a remarkable stenosis at the lower segment of the right ureter, attributed to metastases from her breast carcinoma. We report this case with the aim to make both oncologists and urologists aware of this rare condition.

## 1. Introduction

Globally, breast cancer (BC) is the most common malignancy in women (excluding skin), with over 1.5 million new cases diagnosed annually worldwide, of whom 10–15% are triple negative breast cancer (TNBC) [1]. This malignancy metastasizes mainly to lung, bones, liver, and pleura, being extremely rare to metastasize to the ureter [2,3,4].

Here, we present a patient with a pathologically confirmed TNBC tumor which metastasized to the ureter seven years after the initial diagnosis, and we discuss the diagnostic and therapeutic management.

## 2. Case Report

An 87-year-old woman presented to the Emergency Department at the University Hospital of Patras, Greece, due to an episode of upper gastrointestinal bleeding. Gastroscopy was performed and revealed diaphragmatic hernia complicated with esophagitis. During her hospitalization, she developed painless macroscopic hematuria while she was hemodynamically stable. 

She had a history of diabetes mellitus, hypothyroidism, hypercholesterolemia, hypoxic spongiotic dermatitis, uterine fibromas, as well as follicular lymphoma Grade 3A in right submandibular lymph node (2012) and breast cancer (2015). For her lymphoma, she was treated with six cycles of R-COP and continued maintenance rituximab treatment for six months, omitting radiotherapy.

**Figure 1:**
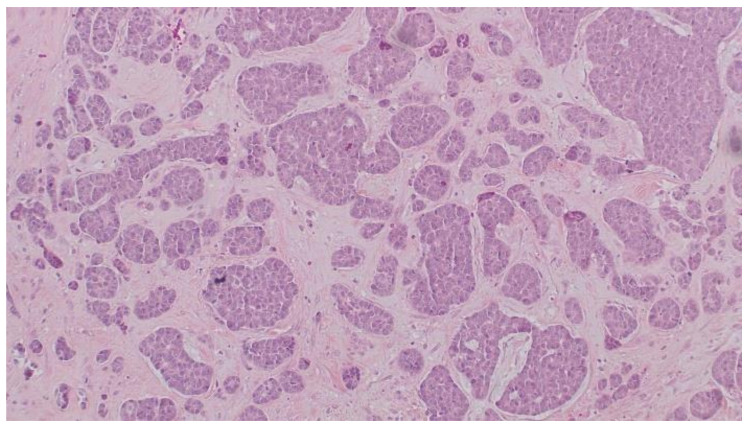
Histology of primary invasive breast carcinoma, nonspecific type (H + E, × 200).

The patient was 80 years old when diagnosed with left breast tumor. Initial mammography revealed a 2.5 cm lesion which was classified as BIRADS V. A partial mastectomy without a sentinel biopsy was performed, because of her age and the negative imaging of axilla. The histopathology revealed in situ and invasive ductal carcinoma of the breast, NST, Grade 2. Immunohistochemistry showed the tumor to be ER (−), PR (−), HER2 (−), and Ki67 (20%), so the diagnosis of a TNBC was confirmed (Figure 1). The tumor was staged as pT2Nx, and the patient received adjuvant chemotherapy (CMF six cycles) and postoperative radiotherapy (Tangential fields, 6MV, 45 Gy).

**Figure 2:**
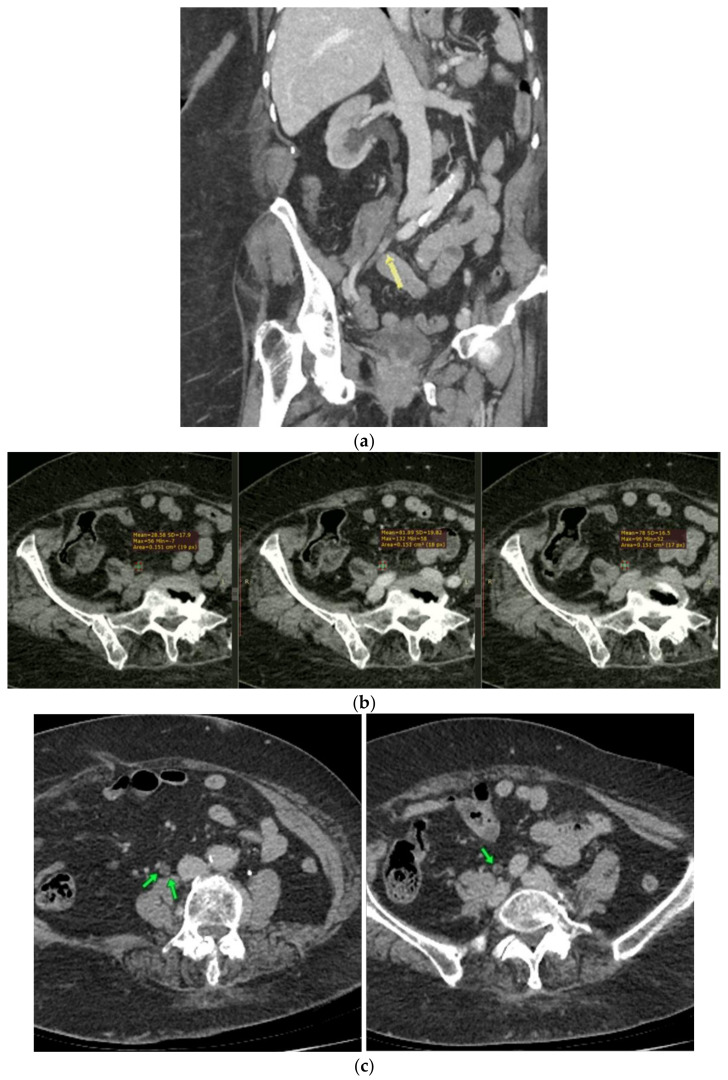
(**a**) Oblique frontal view at the nephrographic phase demonstrating RT kidney obstruction. There is an intraluminal soft tissue mass (arrow) at the mid third of the ureter, causing upstream dilatation of the ureter and ipsilateral pelvicalyceal system. (**b**) Axial images at the level of the ureteral obstruction. Pre- and post-contrast phases, showing the strong and prolonged enhancement of the intraluminal mass. (**c**) Axial images in the nephrographic phase. Arrows indicate some tiny spots of enhancement along the wall of the dilated upper part of the right ureter.

During her admission, the hematuria was investigated both radiologically and endoscopically. Laboratory examinations were normal, urine cytology revealed increased number of RBCs, while urine culture was negative, and serum tumor markers were within normal limits (CEA, CA 15-3, CA 19-9, CA 125). A CT-Urography was performed and showed the presence of a soft tissue mass at the mid third of the right ureter. The mass was 18mm long, completely intraluminal, presenting strong enhancement at the nephrographic and delay phase. Some tiny spots of enhancement were also evident at the upper part of the dilated ureter. No stones or extraluminal mass were found. Head of the differential diagnosis was the multifocal urothelial cancer, but histology was deemed necessary due to her history of breast and lymphoma (Figure 2a–c).

A ^18^F-FDG PET/CT was performed, and no hypermetabolic lymph nodes were detected either above or below the diaphragm. The already known lesion of the right ureter was not visible on PET due to the small size of the lesion and the presence of radioactive urine at the area of interest.

**Figure 3:**
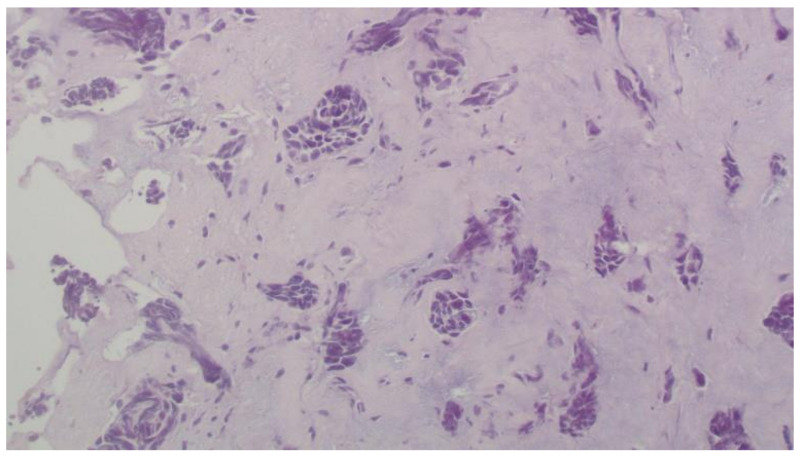
Metastatic carcinoma of the ureter (H + E, × 200).

**Figure 4:**
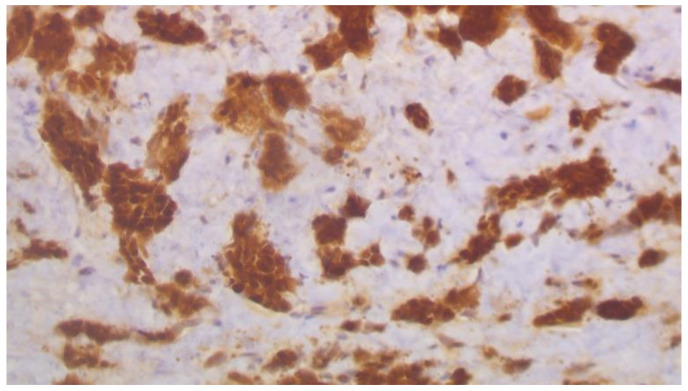
SOX10 immunohistochemical expression in metastatic carcinoma shows strong nuclear staining in 80% of tumor nuclei (×200).

**Figure 5:**
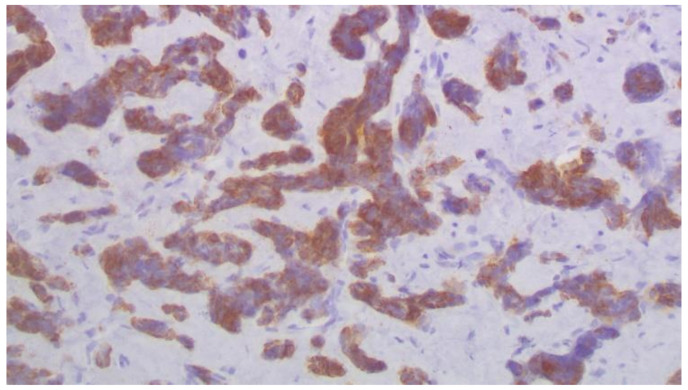
CK7+ immunohistochemical expression in metastatic carcinoma (×200).

Cystoscopy was performed, a pigtail was inserted, and a biopsy from the ureter was taken. Histopathology revealed infiltration of the ureter by tumor cell aggregates morphologically similar to the triple negative breast cancer diagnosed in 2015. Immunohistochemistry showed positivity of cancer cells for SOX10, CK7, and GATA3, and negativity for ER, PR, and HER2 (Figure 3, Figure 4 and Figure 5).

The final diagnosis was ureteral metastasis from the reported triple-negative breast cancer, appearing six years after the initial diagnosis.

**Figure 6:**
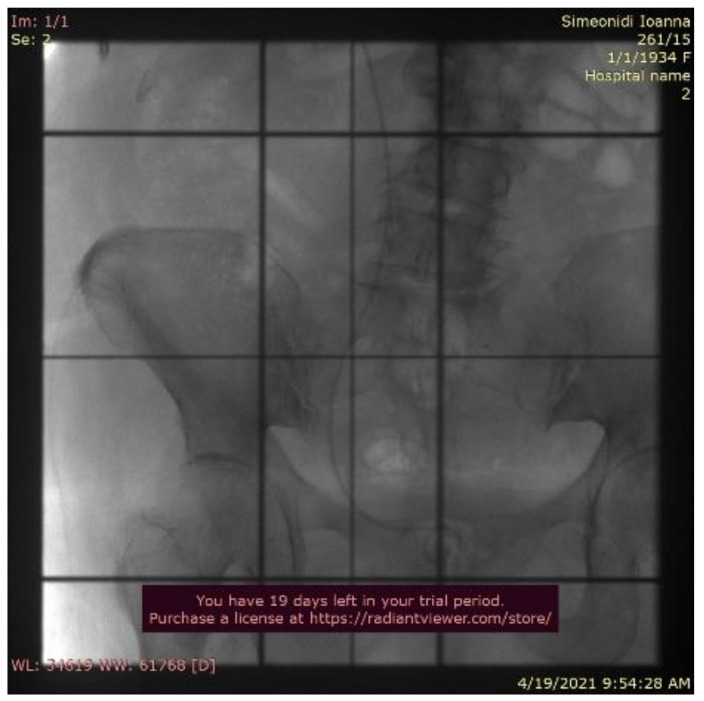
Simulation film.

Even though the ureter was the only site of metastatic disease, due to her age and serious comorbidities, it was decided to treat the metastatic lesion with external beam radiotherapy instead of surgery. A total dose of 37.5 Gy was given to the right ureter with two parallel opposed fields in 15 fractions, using a linear accelerator of 6 MV energy, after simulation (Figure 6).

The patient had an uneventful post-treatment course and 12 months since the completion of radiotherapy she remains in complete clinical remission.

## 3. Discussion

Ureter remains a rare anatomical site for metastases from any primary tumor. In the period 1909–1999, a total of 342 cases of ureteral metastases were collected and reviewed by Haddad et al. [5,6]. The authors found that stomach, prostate, and bladder tumors have a higher tendency to develop ureteral metastases and breast carcinoma, accounting for 7.8% of all cases.

Several case reports have reported on the incidence of this entity. A study of 215 autopsies from patients with breast cancer revealed 42 cases (19.5%) of ureteral metastases [7]. It was stated that the chance to diagnose ureteral metastases in patients while they are alive is remarkably rare [8,9]. In the past twenty years, a very limited number of new cases from patients with solid tumors or hematologic malignancies were reported [10,11,12,13,14,15,16]. A limitation to these studies is that most of them did not report autopsy findings and predated the wide use of computing tomography.

It is difficult to distinguish on clinical and imaging data metastatic ureteral tumors from primary ureteral urothelial carcinoma. Cytology specimens are of limited help, as metastatic ureteral tumors do not adhere to the mucosa. Presman and Ehrlich described in 1948 two cases of ureteral metastases and made important pathologic observations. Metastases were present in all levels of the ureter, were usually bilateral, and had the appearance of either localized nodules or diffuse infiltrating type causing frequently occlusion of the ureteral lumen. The mucosa was usually intact, except in patients with hematuria [17]. 

In most cases, ureteroscopy reveals a stenosis of the ureter. To obtain an accurate diagnosis, biopsy under ureteroscopy is regarded as the most reliable procedure, considering that occasionally the limited tissue sample may attenuate the pathological diagnosis. Exploratory laparotomy is recommended, followed by excision of the metastatic lesion.

An up-to-date literature review has shown that our case is the third one, published on TNBC, presented with invasion of the ureter; one case being a male patient [18,19] (Table 1). ER+/PR−; HER2+, and ER+/PR+; HER2+ breast malignancies have also been reported [13].

A possible explanation for the rarity of this condition can be either the fact that TNBC has a higher rate of recurrences and distant metastases leading to poorer prognosis, or that if there is no hematuria or obstructive uropathy present, the lesions escape from the attention of the physicians.

Another important observation made in our case was that the patient was diagnosed with lymphoma and three years later with TNBC. Molecular biology analysis has shown that between lymphomas and TNBC, the catalytic subunit of the polycomb repressor complex EZH2 inhibition stands out. In diffuse large B-cell lymphomas, inhibition of EZH2 was shown to enhance tumor cell antigen presentation [20], while expression of the same molecule promotes the formation of TNBC in transgenic mice [21].

Imaging modalities such as CT, MRI, and PET/CT are necessary for diagnostic purposes, but they are not specific for metastatic ureteral tumors [22,23]. In our case, we applied a positron emission tomography/computed tomography (PET/CT) scan with poor results regarding the identification of the metastatic lesion.

Despite the wide use of (18)F-FDG PET/CT in modern oncology, in urological oncology, this imaging technique has shown limited value due to the low uptake and to the excretion of FDG via the urine. The consequence of this limitation is that there is a lack of relevant studies stressing the diagnostic value of PET/CT aw a single diagnostic modality [22,24,25].

Although a cure for metastatic breast cancer is lacking, a multidisciplinary, personalized approach can offer symptomatic treatment, relieving symptoms and improving the quality of life of these patients [26,27,28]. Particularly, patients presenting with urinary tract obstruction are candidates for local treatments such as placement of ureteral stents, percutaneous nephrostomy, and external beam radiotherapy [14].

For our patient, transurethral biopsy followed by placement of a pigtail stent and external radiotherapy was considered a better solution than the ureterectomy followed by chemotherapy.

## 4. Conclusions

We report a case of ureteral metastases in a patient with TNBC. We present the non-specific symptoms and the limitations of the available imaging methods in obtaining a diagnosis. Endoscopic procedures are important in identifying the metastatic site and performing a biopsy. The therapeutic procedures should be individualized and based on the histopathology of the lesion and the current state of the patient. A multidisciplinary team of urologists, radiologists, and oncologists should address the necessary diagnostic and therapeutic approach.

## Figures and Tables

**Table 1:** Summary of patients’ characteristics in published cases.

Author and Year {Ref}	Patient’s Characteristics	Clinical Findings	Laboratory Findings	Treatment
Chahin et al., 2020 [18]	54-year-old female Invasive lobular carcinoma, G I, ER-PR-HER2-negative	Hydronephrosis	CT scan: Hemi-pelvic mass	Ureteral stent
Chen et al., 2021 [19]	60-year-old male Invasive ductal carcinoma, GIII, ER-PR-HER2-negative	Hematuria	CT scan: Ureteral mass and dilated ureter	Middle ureter dissection and anastomosis

## Data Availability

The data presented in this study are available on request from the corresponding author.

## References

[1] Lei S., Zheng R., Zhang S., Wang S., Chen R., Sun K., Zeng H., Zhou J., Wei W. (2021). Global Patterns of Breast Cancer Incidence and Mortality: A Population-Based Cancer Registry Data Analysis from 2000 to 2020. Cancer Commun..

[2] Yamamura J., Kamigaki S., Fujita J., Osato H., Manabe H., Tanaka Y., Shinzaki W., Hashimoto Y., Komoike Y. (2021). New Insights into Patterns of First Metastatic Sites Influencing Survival of Patients with Hormone Receptor-Positive, HER2-Negative Breast Cancer: A Multicenter Study of 271 Patients. BMC Cancer.

[3] Nathanson S.D., Detmar M., Padera T.P., Yates L.R., Welch D.R., Beadnell T.C., Scheid A.D., Wrenn E.D., Cheung K. (2021). Mechanisms of Breast Cancer Metastasis. Clin. Exp. Metastasis.

[4] Hu J., Deng J., Guo J., Fu B. (2019). Ureteral Involvement by Metastatic Malignant Disease. Clin. Exp. Metastasis.

[5] Haddad F.S. (1999). Metastases to the Ureter. Review of the World Literature, and Three New Case Reports. J. Med. Liban..

[6] López-Martínez R.A., Stock J.A., Gump F.E., Rosen J.S. (1996). Carcinoma of the Breast Metastatic to the Ureter Presenting with Flank Pain and Recurrent Urinary Tract Infection. Am. Surg..

[7] Grabstald H., Kaufman R. (1969). Hydronephrosis Secondary to Ureteral Obstruction by Metastatic Breast Cancer. J. Urol..

[8] Talreja D., Opfell R.W. (1980). Ureteral Metastasis in Carcinoma of the Breast. West. J. Med..

[9] Richie J.P., Withers G., Ehrlich R.M. (1979). Ureteral Obstruction Secondary to Metastatic Tumors. Surg. Gynecol. Obstet..

[10] Karaosmanoglu A.D., Onur M.R., Karcaaltincaba M., Akata D., Ozmen M.N. (2018). Secondary Tumors of the Urinary System: An Imaging Conundrum. Korean J. Radiol..

[11] Fröber R. (2007). Surgical Anatomy of the Ureter. BJU Int..

[12] Gabsi A., Yahiaoui Y., Zenhani A., Herbegue K., Meddeb K., Mokrani A., Letaief F., Ayadi M., Rais H., Chraiet N. (2018). Ureteral Metastasis in Carcinoma of the Breast. Urology case reports. November.

[13] Jani K. (2006). Ureteric Obstruction Secondary to Metastatic Breast Carcinoma. Pakistan J. Med. Sci..

[14] Logothetis C., Assikis V., Sarriera J., Kufe D.W., Pollock R.E., Weichselbaum R.R., Best R.C., Gansler T.S., Holland J.F., Frei E. (2003). Algorithm for the Management of Urinary Obstruction. Holland-Frei Cancer Medicine.

[15] Merchan J., Jhaveri K. Chemotherapy Nephrotoxicity and dose Modification in Patients with Kidney Impairment: Conventional Cytotoxic Agents-UpToDate. https://www.uptodate.com/contents/chemotherapy-nephrotoxicity-and-dose-modification-in-patients-with-kidney-impairment-conventional-cytotoxic-agents#H1991902678.

[16] Numakura K., Tsuchiya N., Obara T., Tsuruta H., Saito M., Narita S., Inoue T., Horikawa Y., Satoh S., Habuchi T. (2011). A Case of Ureteral Malignant Lymphoma Diagnosed by Laparoscopic Needle Biopsy. Jpn. J. Clin. Oncol..

[17] Presman D., Ehrich L. (1948). Metastatic Tumors of the Ureter. J. Urol..

[18] Chahin M., Chhatrala H., Krishnan N., Brow D., Zuberi L. (2020). Triple-Negative Lobular Breast Cancer Causing Hydronephrosis. J. Investig. Med. High Impact Case Rep..

[19] Chen Y., Wu J., Hu T., Wang J., Su F. (2021). Male Breast Cancer with Ureteral Metastasis: A Case Report. Annals of palliative medicine. China July.

[20] Ennishi D., Takata K., Béguelin W., Duns G., Mottok A., Farinha P., Bashashati A., Saberi S., Boyle M., Meissner B. (2019). Molecular and Genetic Characterization of MHC Deficiency Identifies EZH2 as Therapeutic Target for Enhancing Immune Recognition. Cancer Discov..

[21] Nie L., Wei Y., Zhang F., Hsu Y.-H., Chan L.-C., Xia W., Ke B., Zhu C., Deng R., Tang J. (2019). DK2-Mediated Site-Specific Phosphorylation of EZH2 Drives and Maintains Triple-Negative Breast Cancer. Nat. Commun..

[22] Tanaka H., Yoshida S., Komai Y., Sakai Y., Urakami S., Yuasa T., Yamamoto S., Masuda H., Koizumi M., Kohno A. (2016). Clinical Value of ^18^F-Fluorodeoxyglucose Positron Emission Tomography/Computed Tomography in Upper Tract Urothelial Carcinoma: Impact on Detection of Metastases and Patient Management. Urol. Int..

[23] Wan X., Hou Y., Yu Z. (2010). Helical CT Diagnosis of the Primary Ureteral Carcinoma and Ureteral Metastatic Carcinoma. J. Clin. Radiol..

[24] Huang Y., He H., Wei W., Li Q., Long X., Li Y., Chen R., Yi X. (2021). 18F-FDG PET/CT Features of Ureteral Metastases from Breast Cancer: A Case Report. J. Int. Med. Res..

[25] Kitajima K., Yamamoto S., Fukushima K., Minamimoto R., Kamai T., Jadvar H. (2016). Update on Advances in Molecular PET in Urological Oncology. Jpn. J. Radiol..

[26] Peart O. (2017). Metastatic Breast Cancer. Radiol. Technol..

[27] Lin N.U., Thomssen C., Cardoso F., Cameron D., Cufer T., Fallowfield L., Francis P.A., Kyriakides S., Pagani O., Senkus E. (2013). International Guidelines for Management of Metastatic Breast Cancer (MBC) from the European School of Oncology (ESO)-MBC Task Force: Surveillance, Staging, and Evaluation of Patients with Early-Stage and Metastatic Breast Cancer. Breast.

[28] Pagani O., Senkus E., Wood W., Colleoni M., Cufer T., Kyriakides S., Costa A., Winer E.P., Cardoso F. (2010). International Guidelines for Management of Metastatic Breast Cancer: Can Metastatic Breast Cancer Be Cured?. J. Natl. Cancer Inst..

